# Linguistic issues behind visual question answering

**DOI:** 10.1111/lnc3.12417

**Published:** 2021-06-04

**Authors:** Raffaella Bernardi, Sandro Pezzelle

**Affiliations:** ^1^ CIMeC and DISI University of Trento Trento Italy; ^2^ ILLC University of Amsterdam Amsterdam The Netherlands

## Abstract

Answering a question that is *grounded* in an image is a crucial ability that requires understanding the question, the visual context, and their interaction at many linguistic levels: among others, semantics, syntax and pragmatics. As such, visually‐grounded questions have long been of interest to theoretical linguists and cognitive scientists. Moreover, they have inspired the first attempts to computationally model natural language understanding, where pioneering systems were faced with the highly challenging task—still unsolved—of jointly dealing with syntax, semantics and inference whilst understanding a visual context. Boosted by impressive advancements in machine learning, the task of answering visually‐grounded questions has experienced a renewed interest in recent years, to the point of becoming a research sub‐field at the intersection of computational linguistics and computer vision. In this paper, we review current approaches to the problem which encompass the development of datasets, models and frameworks. We conduct our investigation from the perspective of the theoretical linguists; we extract from pioneering computational linguistic work a list of *desiderata* that we use to review current computational achievements. We acknowledge that impressive progress has been made to reconcile the engineering with the theoretical view. At the same time, we claim that further research is needed to get to a unified approach which jointly encompasses all the underlying linguistic problems. We conclude the paper by sharing our own desiderata for the future.

## 1 INTRODUCTION

Anyone interested in studying language has to deal with a core aspect of it: ‘meaning’. If one wants to understand how meaning is acquired by children or how it can be interpreted by a computer, the question of how it can be represented arises and, with it, sooner or later the importance of *grounding* meaning representations into the visual context pops up. From Quine (1960) to Barsalou (2008), convincing arguments have been made to highlight the importance of developing models able to understand what a word (a symbol) refers to, namely models that account for the symbol grounding problem (Harnad, 1990; Searle, 1980).

‘*What is the representation of a zebra? It is just the symbol string “horse & stripes”. But because “horse” and “stripes” are grounded in their respective iconic and categorical representations, “zebra” inherits the grounding, through its grounded symbolic representation. In principle, someone who had never seen a zebra (but had seen and learned to identify horses and stripes) could identify a zebra on first acquaintance armed with this symbolic representation alone (plus the nonsymbolic – iconic and categorical – representations of horses and stripes that ground it)*’ (Harnad, 1990, p. 343).

Through the years, various proposals have been made to tackle this challenge. Based on different frameworks, they link computational models of language with computational models of vision (Baroni, 2016; Bisk et al., 2020; Jackendoff, 1987; Landauer & Dumais, 1997; Silberer et al., 2017).

Another well‐established core claim about language shared across disciplines is that language is a process of communication (Carpenter et al., 1998; Fazly et al., 2010; Winograd, 1972).

‘*[S]ocial processes [...] make language acquisition possible by creating a shared referential framework within which the child may experientially ground the language used by adults*’ (Carpenter et al., 1998, p. 24).

A crucial role in interaction is played by question‐answer exchanges. Questions have attracted the interest of theoretical linguists who have studied, for instance, how their syntactic structure helps build their meaning (for an overview see, e.g., Borsley & Müller, 2019), or formal semanticists who have studied the role played by the answer to build the truth functional meaning of the question (for an overview, see Groenendijk & Stokhof, 1997; Wisniewski, 2015). More recently, empirical studies have been carried out on how the comprehension of questions emerges, showing that children learn to understand *wh‐* questions before learning information oriented polar questions. The motivation has the root in the assumption that question understanding emerges as a consequence of interactive learning (Moradlou et al., 2021).

**FIGURE 1:**
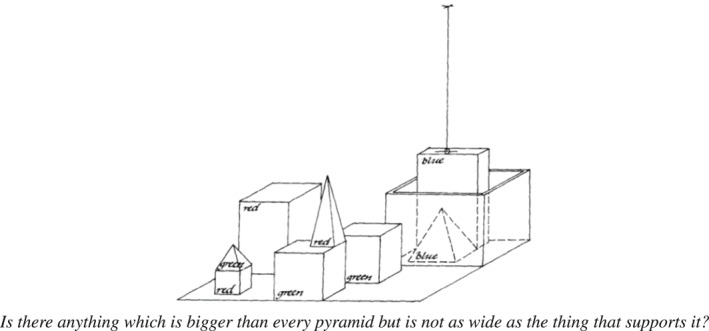
One visually‐grounded question in Winograd (1972). To answer it, the system has to handle reasoning abilities and deal with language ambiguity, vagueness, negation and pragmatics, viz., the list of desiderata we extract from Winograd’s detailed dialogue sample

The importance of combining these two main challenges—modelling of symbolic grounding and communication exchanges—was acknowledged by one of the first computational systems about natural language understanding, which focused on visually‐grounded dialogues. Winograd (1972) introduced a system that ‘answers questions, executes commands, and accepts information in an interactive English dialog’ (Winograd, 1972, p. 1). Crucially, such questions are about a visual scene, illustrated in Figure 1: it contains a table on which there are several boxes and pyramids; a person gives instructions, related to such scene, to a robot which has to execute them (e.g., ‘pick up a big red block’). The step of obtaining a representation of the visual input was put aside—the system was fed with a pre‐compiled symbolic representation of the scene—with the focus being on language understanding. Winograd provided a detailed dialogue sample to discuss the various functionalities such a system must simultaneously deal with at various linguistics levels, namely syntax, semantics and inference. The system had to be able to deal with questions containing an anaphoric expression; to draw inference beyond the question necessary to give the answer; to ask to clarify ambiguity. It was expected to be able to understand when it did not understand the question; when it did not know the answer; when the question was non‐sense. Furthermore, questions were grounded into the scene as well as in the language context, hence they had to be interpreted and answered based on the previous dialogue history. For instance, to answer the question given as example in Figure 1, the system had to reason on relations between sets of objects (‘anything *bigger than* every pyramid’), interpret negation (‘but is *not* as wide’) and resolve the anaphora (‘that supports *it*’).

From Winograd’s sample dialogue, we extract a list of main linguistic phenomena (ambiguity, vagueness, negation) and skills (reasoning and pragmatic‐based interpretation) that we believe a multimodal system should be able to model. We will refer to this list as our *desiderata*, that we use to review recent achievements in a specific subtask tackled by Winograd’s system, namely answering visually grounded questions.

Thanks to efforts within computational linguistics and computer vision, Visual Question Answering (VQA) has become a widely studied task, and important progress has been made on the development of computational multimodal models. VQA has been treated both as a downstream task and as a pre‐training task to effectively encode multimodal input and trasfer it to other multimodal tasks. In this paper, we will discuss both uses of it. By reviewing how current models handle each of our desiderata, we will highlight where further research is needed to turn VQA from an in‐lab exercise to a real‐life application, and point to what we think is feasible to achieve in the short and medium term.

## 2 THE RECENT REVIVAL OF VQA

In the last years, there has been a steep increase of interest in the task of answering visually grounded questions. This revival was motivated by the development of models to assist visually impaired people (Bigham et al., 2010) or the attempt to establish a Turing Test based on visual information (Malinowski & Fritz, 2014). This pioneering work was immediately followed by a vigorous worldwide effort aimed at building new datasets and models (Antol et al., 2015; Gao et al., 2015; Geman et al., 2015; Goyal et al., 2016, 2017; Malinowski et al., 2015; M. Ren, Kiros et al., 2015; Yu et al., 2015). This effort has been exhaustively summarized in various surveys (Kafle & Kanan, 2017b; Manmadhan & Kovoor, 2020; Srivastava et al., 2021; Wu et al., 2017), as well as tutorials (Kordjamshidi et al., 2020; Teney et al., 2017).^1^ In particular, Srivastava et al. (2021) nicely sketch the timeline of the major breakthroughs in VQA in the last five years, whilst Wu et al. (2017) provide interesting connections with structured knowledge base and an in‐depth description of the question/answer pairs present in VQA datasets. Finally, Kafle and Kanan (2017b) discuss shortcomings of current VQA datasets.

### 2.1 The VQA task

**FIGURE 2:**
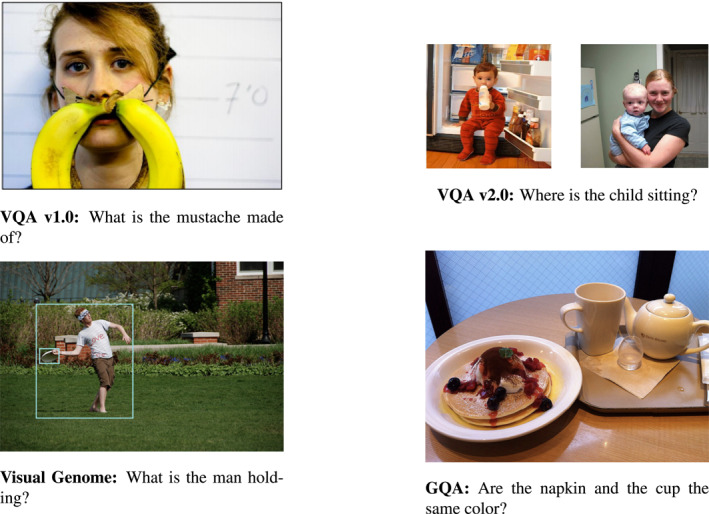
Datasets of natural images: The task of answering a question about an image has been promoted by the release of datasets containing an image and a question about it, such as VQA v1.0 (Antol et al., 2015). By controlling for the multimodal data points, models have been pushed to build *finer‐grained representations* (see VQA v2.0; Goyal et al., 2017). The release of densely annotated datasets, such as Visual Genome (Krishna et al., 2017), made it possible to tackle the challenge of building multimodal representations of *relations between objects*. This paved the way to resources, such as GQA (Hudson & Manning, 2019), which include compositional questions involving such relations

Since 2015, the VQA challenge is organised yearly. Thanks to it, progress in the field can be constantly monitored.^2^ The original dataset (VQA v1.0) consisted of images taken from the Microsoft Common Objects in Context (MS‐COCO) dataset (T. Y. Lin et al., 2014) and questions collected from human annotators via crowdsourcing. As we will discuss later, the baseline model relied on coarse multimodal representations obtained by performing simple operations on the language and visual representations. The original dataset was shown to contain heavy biases that models could easily exploit to perform the task (B. Zhou et al., 2015). Since then, quite some attention has been paid to the language bias issue. In particular, a new dataset has been released (VQA v2.0; Goyal et al., 2017) in which each question is paired with very similar images. Figure 2 illustrates the difference compared to the previous version of the VQA dataset, a change that requires *finer‐grained representations* as it was advocated by, for example, Shekhar et al. (2017) and J. Wang et al. (2018).

Since the original work on VQA, a careful analysis of model results has been carried out to go beyond an evaluation solely based on task success (see, for instance, Agrawal et al., 2016; X. Lin & Parikh, 2015; Zhu et al., 2016). Driven by the goal of gaining a deep understanding of the multimodal behaviour, Agrawal et al. (2018) reorganised the VQA dataset to assess the robustness of models when exposed to different question biases at test time compared to what is seen during training. From these analyses, it turned out that questions involving *reasoning about relations between objects*, such as, for instance, those involving role labelling and spatial relations, are the hardest to be answered. To help making progress on questions involving role labelling, Yatskar et al. (2016) released ImSitu, a dataset containing annotation about actions, roles and objects. In parallel, datasets such as Visual7W (Zhu et al., 2016), TDIUC (Kafle & Kanan, 2017a), Visual Genome (Krishna et al., 2017) and GQA (Hudson & Manning, 2019) have been developed to test the visual reasoning and compositionality abilities of models. Figure 2 (bottom) illustrates the Visual Genome and GQA datasets by means of example. The main novelty of Visual Genome is the high‐density annotation of its images and the fine‐grained alignment between images and language descriptions. The GQA dataset was carefully designed building on such annotation (Hudson & Manning, 2019). By adopting a *diagnostic* approach, they paired natural images of Visual Genome with automatically‐generated questions to enable a fine‐grained diagnosis for different question types. Furthermore, they introduced new metrics aimed to evaluate models with respect to consistency, plausibility and grounding. Finally, both for VQA and GQA new out‐of‐domain test sets have been proposed to allow a more reliable evaluation of the models (Gokhale et al., 2020a; Kervadec et al., 2021).

**FIGURE 3:**
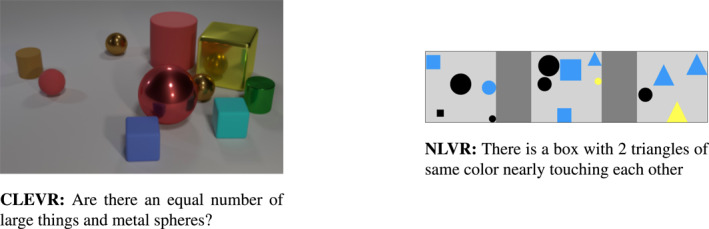
Datasets of synthetic images: Benchmarks like CLEVR (Johnson, Hariharan, van der Maaten, Fei‐Fei, et al., 2017) and NLVR (Suhr et al., 2017) require models capture the relations between the objects depicted in a synthetic scene. In CLEVR, relations involve objects depicted in a single scene; in NLVR, they span over three *boxes*. Language is synthetically generated in CLEVR and crowd sourced in NLVR

The diagnostic approach has been undertaken also in other work proposing datasets of synthetic images coupled with either templated (Andreas et al., 2016; Johnson, Hariharan, van der Maaten, Fei‐Fei, et al., 2017; Kuhnle & Copestake, 2017; Sorodoc et al., 2016; Zhang et al., 2016) or crowd sourced (Suhr et al., 2017) language. Figure 3 illustrates samples from CLEVR (Johnson, Hariharan, van der Maaten, Fei‐Fei, et al., 2017) and NLVR (Suhr et al., 2017). CLEVR images are paired with questions generated through functional programs; the data points are carefully designed to test the skills a model needs to master to answer attribute, existential and counting questions, as well as questions based on comparisons and spatial relationships. NLVR (Suhr et al., 2017) is based, instead, on a verification task: models have to answer whether a given sentence is true or false within the given visual context. This is the same setting of NLVR2 (Suhr et al., 2019), which uses natural images. In Section 3, we will come back to the role of diagnostic datasets to evaluate the reasoning abilities of multimodal models.

From the very beginning of this VQA revival, attention has been paid also to questions that require information available in a Knowledge Base to be answered (Wang et al., 2017a). This line of research has been pursued by several studies, particularly thanks to the introduction of the Fact‐based VQA dataset (Wang et al., 2017b). The VQA task is now taking new directions, such as embodied approaches where an agent has to navigate an environment and answer questions about it (H. Chen et al., 2019; Das et al., 2018); video VQA, where the answer has to be found in videos rather than in static images (Lei et al., 2018, 2020); answering questions about diagrams and charts (Ebrahimi Kahou et al., 2017; Kafle et al., 2018); text VQA, which involves recognizing and interpreting textual content in images (Biten et al., 2019; Han et al., 2020); answering questions about medical images (see, Abacha et al., 2020); and many others.

### 2.2 Multimodal representations

Research on the interplay between language and vision has benefited from the comparable representations developed and used by the computational linguistics and computer vision communities in the last decade or so. On the language side, the distributional semantics approach (Firth, 1957; Harris, 1954) has become the most popular view of natural language semantics: A word is represented by a *vector* (also called *word embedding*) which encodes the contexts in which it occurs (Landauer & Dumais, 1997). In traditional approaches this vector is *static*, that is, not dependent on the various senses of a word (see, Mikolov et al., 2013; Pennington et al., 2014), whilst last‐generation neural network models, such as Transformers, are able to produce *contextualized* representations whilst processing a linguistic string (see, Devlin et al., 2019). Similarly, a whole image (or each of the objects in it) is represented by a vector computed by a deep neural network, which learns such representation in an end‐to‐end fashion, viz. starting from the image’s pixels whilst being trained on an object classification task (He et al., 2016; S. Ren, He, et al., 2015; Simonyan & Zisserman, 2015).

The availability of word embeddings and visual vectors has facilitated the fertilization between the two communities, that has been further boosted by the availability of multimodal baselines and state‐of‐the‐art models. Earlier approaches obtained multimodal representations by concatenating the linguistic and visual vectors (Bruni et al., 2014) or by taking their inner product (Antol et al., 2015).^3^ We are currently experiencing the boom of Transformer‐based *universal multimodal encoders* pretrained on several multimodal tasks, and aimed at obtaining task‐agnostic multimodal representations (Y.‐C. Chen et al., 2020; Li et al., 2019; Lu et al., 2019; Su et al., 2019; H. Tan & Bansal, 2019; L. Zhou et al., 2020).

### 2.3 VQA models

#### 2.3.1 Early models

The most popular VQA baseline model by Antol et al. (2015) learns the word embeddings through the VQA task itself; starting from one‐hot‐encodings, it builds word embeddings that are incrementally composed by an LSTM (Long Short Term Memory; Hochreiter & Schmidhuber, 1997) to obtain the question representation; for the images, it uses VGGNet image embeddings (Simonyan & Zisserman, 2015), which are further processed by a linear transformation to match the LSTM encoding of the question. These features are then combined using element‐wise operations to a common multimodal feature; this is given as input to a softmax classifier to obtain the probability distribution among the candidate answers, and select the one with the highest probability. Building on this early VQA baseline, a plethora of models have been proposed. Since exhaustive overview papers are already available (Kafle & Kanan, 2017b; Manmadhan & Kovoor, 2020; Srivastava et al., 2021; Wu et al., 2017), here we do not review all the approaches and models that have been proposed. Instead, we highlight and explain the major milestones that have been achieved and that we can relate to our desiderata listed in Section 1.

#### 2.3.2 Attention‐based models

The first crucial enhancement has been the use of *attention mechanisms* which have led to build *fine‐grained* representation of the multimodal input. One modality guides the interpretation of the other so to give more weight to salient regions of the image or to relevant words of the question (Yang et al., 2016). The promising results obtained with the introduction of these reweighting methods led researchers to propose more complex mechanisms like hierarchical co‐attention (Lu et al., 2016), or the combination of bottom‐up and top‐down mechanisms, an approach that has dominated the scene since its introduction by Anderson et al. (2018). A detailed analysis of the effect of its various parameters is given by Teney et al. (2018). The main advancement brought by the bottom‐up top‐down approach lies in the use of attention to focus on the objects in the scene that are most salient to answer the question—rather than on generic (important) regions of the image. This is made possible by the use of Faster R‐CNN (S. Ren, He, et al., 2015), which proposes several candidate bounding boxes—each containing one object—to the network. The object identification phase allows the model to exploit bottom‐up information regarding objects instead of starting from scratch from the understanding of the entire scene, and informs the top‐down component which selects the relevant objects to perform the task.

#### 2.3.3 Neural module networks

This family of models treats a question as a collection of predefined subproblems (e.g., counting, localization, conjunction, etc.), each handled by a dedicated module. Whilst NMN (Andreas et al., 2016) requires a parser to process the sentence into its components, N2NMN (Hu et al., 2017) does not require any external supervision. Building on these approaches, hybrid methods which combine symbolic and neural components have been recently promoted. Johnson, Hariharan, Van Der Maaten, Hoffman, et al. (2017) claim that models based only on neural representations unavoidably learn dataset biases instead of the visual reasoning skills needed to properly perform complex tasks such as VQA. Hence, they propose a model that represents a question like a program and answers the question by composing functions from a function dictionary. The model learns compositional reasoning from a small amount of the ground‐truth programs used in CLEVR (Johnson, Hariharan, van der Maaten, Fei‐Fei, et al., 2017) to generate the questions. The model is shown to generalize to novel questions by composing modules in ways that are not seen during training. This hybrid approach has been pushed forward by Yi et al. (2018), who propose a neural‐symbolic VQA approach that disentangles reasoning from visual perception and language understanding, and by Mao et al. (2019), who add a neuro‐symbolic concept learner. The hybrid approach does not fall into the bias traps and is easily interpretable—which makes it potentially different from ‘black‐box’ neural networks models.

#### 2.3.4 From labs to real‐life applications

A crucial challenge all these models have to face is the ability to generalize the knowledge learned to unseen data, which can be achieved only if the model is able to compositionally build the multimodal representations, a must for any model of human intelligence (Lake et al., 2017). Since neural‐based VQA models have been shown to produce inconsistent answers to questions that are either similar or mutually exclusive, approaches to mitigate this behaviour have been recently proposed (Ray et al., 2019; Selvaraju et al., 2020). Interestingly, Gandhi and Lake (2020) showed that whilst children are driven by the mutual exclusivity assumption in their learning process, neural networks are not, and set this as an open challenge.

All the work we have reviewed so far has paved the way toward incorporating challenging linguistic phenomena into the VQA framework and benchmarks. However, none of them jointly account for the whole range of phenomena encountered in real‐life question answering scenarios. Once we move from labs to real‐life applications, indeed, additional challenges emerge both at the visual and language level. Models are required to master a variety of language phenomena, such as language ambiguities, pragmatic aspects and context dependence, negation, entailment, mutual exclusivity and all the reasoning skills subtending them. Some of these extra challenges are present in goal‐oriented datasets such as VizWiz (Gurari et al., 2018), which contains pictures taken by visually‐impaired people with their mobile phone, the questions they ask about these pictures, and the corresponding answers provided by human assistants via crowdsourcing.

In the following, we highlight what has been achieved (and what has not) of our list of desiderata extracted from Winograd’s dialogue sample. By so doing, we also emphasize what we believe deserves further attention from the language and vision community.

## 3 REVISITING THE WISHES FROM THE PAST

As we mentioned above, Winograd looked at the challenges for a visually grounded interactive system solely from the perspective of the language modality (the images were assigned pre‐compiled symbolic representations). On the other hand, most of the work carried out recently on VQA has been driven by the computer vision community. We are now in the fortunate position to promote a joint view on how the long‐standing theoretical questions about grounded language understanding are addressed by computational models. Hence, in what follows, we review where the current visually‐grounded research stands with respect to the *desiderata* we extracted from Winograd’s dialogue sample.

### 3.1 Reasoning

Winograd called for a system that is able to infer from the visual scene the answer to a question of the type: ‘*Is there anything which is bigger than every pyramid but is not as wide as the thing that* *supports* *it?*’. As seen in Section 2, in the recent past the reasoning skills of multimodal models have been studied both by controlling the reasoning steps that a system has to perform to answer VQA questions and by building datasets that are specifically designed for testing these abilities.

As we have mentioned above, several diagnostic datasets have been released with the aim to assess model abilities to reason over a question grounded in a visual context (hence, *visual reasoning*; Andreas et al., 2016; Johnson, Hariharan, van der Maaten, Fei‐Fei, et al., 2017; Kuhnle & Copestake, 2017; Suhr et al., 2017, 2019). These works brought a shift from *non‐relational* questions, which require reasoning about the attributes of one particular object instance, to *relational* questions (Santoro et al., 2017), which instead require to genuinely reason over the relations between multiple objects depicted in the image. From the computer vision perspective, solving non‐relational questions implies locating an object in an image, that is, paying *attention* to the region of the image which ‘contains’ the object. Relational reasoning problems, in contrast, require models to pay attention on multiple objects in the visual scene, to identify their attributes (colour, size, category, etc.), and to perform a higher‐level reasoning step over this perceptual knowledge. If one views language from a denotational semantic perspective, it becomes clear that the move from non‐relational to relational skills is also crucial to master language phenomena of increasing complexity, for which yet another step is necessary, namely to deal with questions involving *relations between sets of objects*. For instance, to properly represent quantifiers a model has to identify the sets of relevant objects; similarly, gradable adjectives require a comparison of the set of entities against which they are interpreted; negation of, for example, a noun points to the alternative sets of the negated noun (the set of other candidate objects), etc. Answering to questions involving these expressions is therefore a higher‐level problem as compared to first‐level relations and non‐relational questions described above.

**FIGURE 4:**
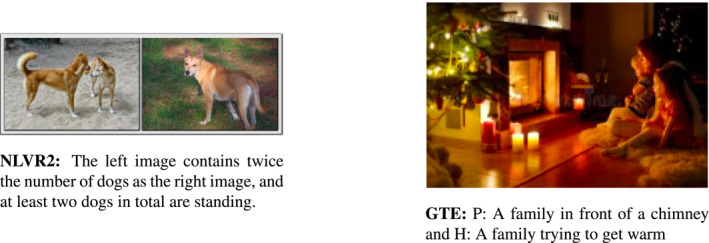
Reasoning: NLVR2 (Suhr et al., 2019) evaluates the high‐level reasoning skill of models: a model has to say whether the given sentence is true or false with respect to the two images; GTE (Vu et al., 2018) evaluates their ability to ground textual entailment: the model has to choose whether the two sentences (a premise P and a hypothesis H) are in an entailment, contradictory or neutral relation, given the image

Recently, the reasoning skills of multimodal models have been tested by means of either probing tasks involving high‐level reasoning or grounded textual entailment (see Figure 4). In the recent NLVR2 dataset (Suhr et al., 2019), a visual scene comprising two natural images is coupled with a crowdsourced statement describing some relation between the entities depicted in these two images. In order to verify whether the statement is true for that scene, models are required to deal with complex linguistic phenomena such as quantification, negation, coreference and syntactic ambiguity resolution. Whilst handling these phenomena is straightforward for humans (who achieve a virtually perfect accuracy in the task), current state‐of‐the‐art models are shown to struggle with them. Indeed, the gap with human performance is around ‐20% in this dataset (see Suhr et al., 2019; Zheng et al., 2020). This reveals that a full understanding of complex language phenomena is an ability required for models to deal with real‐life multimodal questions.

**FIGURE 5:**
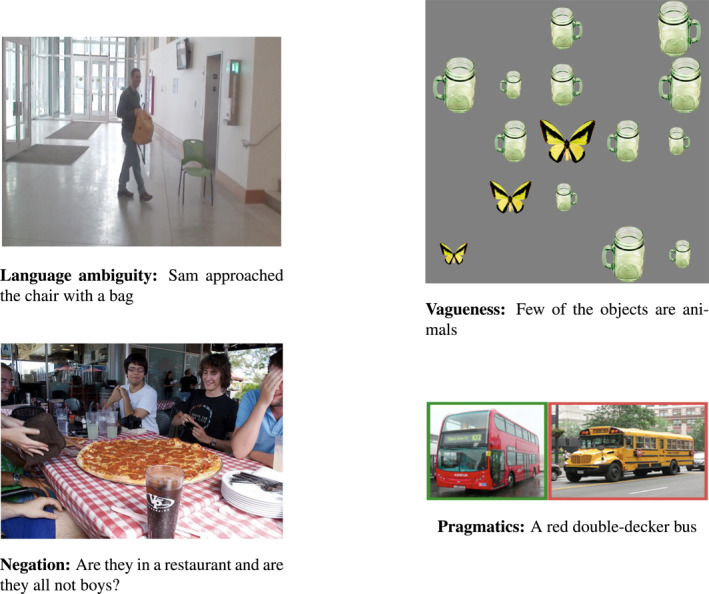
Benchmarks requiring models to compute higher‐level relations, that is, between sets of objects. In Berzak et al. (2015) (top left panel), visual information from videos is used to disambiguate sentences that are ambiguous at many levels, for example, syntactic. Pezzelle et al. (2018) focus on quantifiers and challenge models to learn their vague, context‐dependent interpretation from visual scenes (top right). In Gokhale et al. (2020b), a new dataset and computational method is proposed to tackle negation (bottom left). Finally, Cohn‐Gordon et al. (2018) force Image Captioning models to be pragmatically informative, that is, to produce captions that are discriminative (bottom right)

The reasoning skills of multimodal models have been studied also by directly investigating how they perform on the entailment task. To test these abilities, Vu et al. (2018) proposed a dataset of grounded textual entailment: a model has to say whether two given sentences (a premise and a hypothesis) are in an entailment, contradictory or neutral relation with respect to a given image; whereas Xie et al. (2019) released a visual entailment dataset where models are asked to check whether a given image entails a given text (Figure 5).

### 3.2 Language ambiguity

‘*Put the blue pyramid on the block in the box*’ is one of the instructions the Winograd’s system is challenged to handle. The instruction is syntactically ambiguous, but the visual context disambiguates it. Current multimodal models have been evaluated on the ability to acquire and use such ‘disambiguation’ skills. For example, Christie et al. (2016) addressed the issue of prepositional phrase attachment resolution by training the system to pick, among the possible interpretations produced by a vision and language model, the one that is consistent between the two modalities.

Along with syntactic ambiguities, Winograd’s system is faced with questions and instructions that are ambiguous at the semantic and discourse level since they involve anaphora resolution, for example, ‘*Is it* *supported* *?*’ or ‘*Put a small one into the green cube*’. Berzak et al. (2015) studied ambiguity at the syntactic, semantic and discourse level, and introduced a novel dataset of ambiguous sentences coupled with short videos. Overall, their multimodal model was shown to be able to perform the disambiguation task, with model performance being higher for syntactic compared to either semantic or discourse ambiguities.

Syntax has been shown to be useful also to disambiguate referring expressions and properly locate (ground), in an image, the object to which the expression refers (Cirik et al., 2018). Here, a syntactic analysis of the input referring expression was used to inform the structure of a computation graph. Moreover, some other work (see Shutova et al., 2016) focused on a special type of semantic ambiguity, metaphors and proposed the task of visually‐grounded metaphor detection. Given an adjective‐noun phrase such as ‘black hole’, the task is to understand whether the phrase represents a metaphor or not. Once again, visual information was shown to be useful for the task.

### 3.3 Vagueness

‘*Is at least one of them narrower than the one which I told you to pick up?*’. To answer this and similar questions, Winograd’s system is required to understand quantifiers (*at least one*) and gradable adjectives (here, the comparative form *narrower*). These expressions can be *vague*, that is, their interpretation can depend on the context in which they are used. For example, the applicability of words like *most* or *big* in a certain context depends on the properties of the set of objects that are relevant for their interpretation. Moreover, their interpretation can be *borderline* and therefore differ across human speakers.

Whilst numbers represent a well‐known challenging problem in VQA (Acharya et al., 2019; Chattopadhyay et al., 2017), the presence of quantifiers in standard VQA datasets is limited. Though quantification is present in some visual reasoning benchmarks, such as ShapeWorld (Kuhnle & Copestake, 2017), NLVR (Suhr et al., 2017) and NLVR2 (Suhr et al., 2019), these approaches only include numerical or logical quantifiers, for example, *at least two* or *more than half*. In contrast, quantifiers such as *few* or *most*—whose intepretation largely depends on the (visual) context in which they are uttered—are absent. A strand of work has focused on quantifiers combining formal semantics and cognitive science to propose models to perform grounded quantification in a human‐like manner (Sorodoc et al., 2018); to assign the correct quantifier to a visual scene (Sorodoc et al., 2016); and to model the use of quantifiers jointly with numbers and proportions (Pezzelle et al., 2017, 2018).

Gradable adjectives have long been studied by formal semanticists interested in understanding how word meaning changes depending on the context in which the word is uttered (Kennedy, 2007; Partee, 1995). However, in standard VQA benchmarks, these expressions are treated as static rather than context‐dependent attributes; alternatively, they are present only in their comparative or superlative forms (Kuhnle & Copestake, 2017; Suhr et al., 2017). Recently, Pezzelle and Fernández (2019b) released a novel dataset of synthetically generated images and statements containing the gradable adjectives *big* and *small*, and showed that state‐of‐the‐art visual reasoning models can, to some extent, learn the function underlying their use. However, models were shown to be unable to learn an *abstract* representation of such words that can be compositionally applied to unseen objects (see also Pezzelle & Fernández, 2019a).

### 3.4 Negation

Winograd’s system should also be able to handle negation in order to answer questions like ‘*How many blocks are not in the box?*’. Kruszewski et al. (2016) argue that conversational negation does not create the complement set, but rather the alternative set. If we look at this claim from the perspective of visually‐grounded negation, this means that its interpretation requires looking at the set of alternative entities in the scene, or even understanding that the reference is not in the image (hence, it is not visually grounded). Nordemeyer and Frank (2014) show that processing negation can be easier for humans if a visual context creates pragmatic expectation that motivates its use. However, it is unknown whether this holds for multimodal models. van Miltenburg et al. (2016) provide a preliminary corpus study on the use of negation in image captioning (IC) and points the implication to IC models. Suzuki et al. (2019) propose a logic‐based visual inference system and evaluate it on the retrieval of images from text including logical operators (negation, quantifiers and numerals). More recently, some interest has been paid in the computer vision community to logical skills of VQA models, particularly negation. Gokhale et al. (2020b), for example, showed that state‐of‐the‐art models struggle to handle such phenomenon, and proposed a method and dataset to tackle this problem. Greco et al. (2021) show that multimodal universal encoders have difficulty in interpreting negatively answered questions.

### 3.5 Pragmatics

Winograd’s system is also required to use referring expressions that are pragmatically discriminative based on the context in which they are used; for example, *the big red block* if there are other blocks and none else are both big and red. In the language and vision community, pragmatic aspects have been taken into account in the task of IC, where approaches building on Bayesian frameworks have been proposed to generate descriptions that contrastively refer to one but not another (similar) image (Achlioptas et al., 2019; Andreas & Klein, 2016; Cohn‐Gordon et al., 2018; Monroe et al., 2017). Similar approaches have been proposed for *zero‐shot* referring expression generation (Zarrieß & Schlangen, 2019).

Some recent work investigated the use and interpretation of colour terms in grounded communication contexts. Monroe et al. (2016) focused on the generation of compositional colour descriptions, whilst Monroe et al. (2017) presented a novel corpus of colour descriptions from reference games, and showed that an agent equipped with both a neural listener and speaker component interprets colour descriptions better than the listener alone. More recently, Schüz and Zarrieß (2020) focused on predicting objects’ colours and showed that combining categorical with perceptual, entity‐based information is the best‐performing approach.

## 4 OPEN CHALLENGES AND FUTURE DIRECTIONS

We conclude the survey by touching upon new challenges that we see deserve further attention and could be addressed in the near future.

### 4.1 Further challenges from computational linguistics

As mentioned above, Windograd’s system was designed to ground questions into a visual scene but also based on the dialogue history. The move from a question‐answering system to a QA system able to answer follow‐up questions (FUQs) has been undertaken by the QA community early on. It was shown to be an interesting case‐study in between QA, information retrieval and dialogue systems: users are given the chance to refine their query/question based on the linguistic answer they received (Webb & Webber, 2009). Follow‐up visual questions have been studied for instance in F. Tan et al. (2019), where the system has to retrieve the correct image by receiving a sequence of questions asked by a user.

**FIGURE 6:**
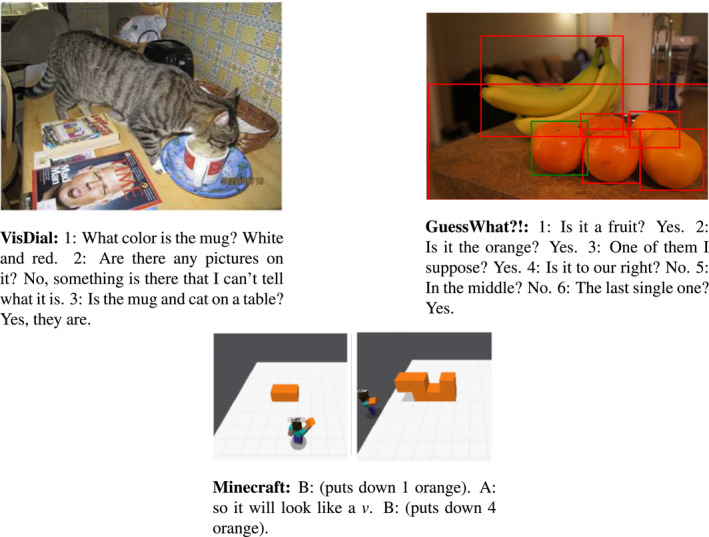
Interactive VQA. In VisDial (Das, Kottur, Gupta, et al., 2017), the model has to ground a follow‐up question into the linguistic and visual context to answer it. GuessWhat?! (de Vries et al., 2017) requires the model to generate a sequence of Y/N‐questions to gather information about the target object to be guessed. Finally, in Minecraft (Jayannavar et al., 2020), a full interaction in the Winograd‐style is required

Multimodal models have been evaluated also on visual dialogue tasks, in which the agent has to answer a FUQ by grounding it on the dialogue history and on the image the question is about. The most popular dataset, VisDial (Das, Kottur, Gupta, et al., 2017), has been used for a yearly organised challenge: a model (the Oracle) has to answer a question about an image given either a caption or a caption together with a sequence of question‐answer pairs about the image (see Figure 6). Agarwal et al. (2020) shows that only 11% of the samples in the VisDial dataset need the previous context to be correctly answered. Hence, this research line requires further effort on the collection of datasets containing more challenging dialogue phenomena. Since universal multimodal encoders are available, checking their grounding skills on the relatively small datasets including dialogue history may be a first interesting step.

When opening the box of interaction, the next challenge that pops up immediately is question generation. Task‐oriented visual games (de Vries et al., 2017; Das, Kottur, Moura, et al., 2017; Haber et al., 2019; Ilinykh et al., 2019) are a good way to measure the progress in such direction. Figure 6 illustrates the simple dialogues of GuessWhat?! game (de Vries et al., 2017). Task success is taken to be a measure of how well the model has been able to ask informative questions. However, as shown by some studies (Mazuecos et al., 2020; Shekhar et al., 2019; Testoni, Shekhar, et al., 2019), task‐success does not relate to the quality of the dialogue nor to the informativeness of the question generated. More work is needed to develop conversational multimodal models that are able to generate pragmatically sound utterances. Crucially, the community lacks datasets to evaluate such skills. An interesting project that could represent an important contribution towards this aim involves two agents playing the Minecraft visual game (Jayannavar et al., 2020).

Most multimodal conversational models exploit the encoder‐decoder architecture (Sutskever et al., 2014): an encoder receives the embeddings of both modalities, it combines them, and uses its hidden state to condition the decoder module to generate the (follow‐up) question. We hope that universal decoders that are able to transfer their knowledge to new tasks will be developed. In line with the general claim advocated by Linzen (2020), we hope to see carefully‐designed visual dialogue datasets that are useful to give exact diagnoses of the communication skills achieved/not yet achieved by the conversational systems.

### 4.2 Further challenges from computer vision

The VQA task has been further extended to QA about videos. The largest‐scale Video‐QA dataset currently available is TVQA (Lei et al., 2018, 2020), which contains questions about popular TV shows. Besides visual grounding, VQA models are also challenged to deal with the audio modality in the Audio‐Visual Scene‐aware Dialogues dataset (AVSD; Hori et al., 2019). Finally, the fervent activities we are experiencing these days on interactive QA over images and videos will certainly create a boost towards the interesting goal of developing models that are able to ‘predict future events’ (Huang et al., 2016; Walker et al., 2014). Humans highly rely on their prediction skills when interpreting a new input, integrating their perceptual signal with prior knowledge. We hope that more awareness of cognitive and neuroscience findings towards the combination of bottom‐up (perceptual) and top‐down (prior) knowledge will help shaping new multimodal models (Schüz & Zarrieß, 2020; Suglia et al., 2020; Testoni, Pezzelle et al., 2019).

## 5 CONCLUSION

Reviewing the literature on VQA is, by itself, a stimulating activity since progress is tangible and fast. We hope that this paper will contribute to promote further work and collaboration between experts in the language and vision community, which we have shown to be crucial for the development of fully fledged multimodal models. We agree with the call for more research on contextual language learning promoted by Bisk et al. (2020), and for the importance of developing a vision and language decathlon benchmark to measure holistic progress advocated by Kafle et al. (2019) (for a first step towards this goal, see Parcalabescu et al., 2020). We furthermore call for more awareness of neuroscience findings on how human brain processes these two modalities and on settings in which the two modalities convey complementary, rather than aligned, information (Pezzelle et al., 2020).

## ENDNOTES

^1^ An updated list of papers can be found here: https://github.com/jokieleung/awesome‐visual‐question‐answering

^2^
https://visualqa.org/

^3^ See Elliott et al. (2016) for an overview.
